# Metaplastic Breast Carcinoma in the Lungs: A Case Report

**DOI:** 10.4021/wjon637w

**Published:** 2014-01-16

**Authors:** Laura Bourdeanu, George Somlo

**Affiliations:** a The Sage Colleges, 65 1st Street, Troy NY 12180, USA; b City of Hope National Medical Center, 1500 East Duarte Road, Duarte CA 91010, USA

**Keywords:** Metaplastic breast cancer, Metastases, Treatment, Prognosis, Pathologic features

## Abstract

Metaplastic carcinoma of the breast is a rare, aggressive form of cancer occurring in less than 1% of all breast cancers. Spindle cell carcinoma is a rare variant of the metaplastic carcinoma seen in less than 10% of such cases. The prognosis of spindle cell breast cancers is poor, hence aggressive treatment with surgery, chemotherapy and/or radiation is required. Here we report a case of a 62-year-old female with metastatic metaplastic breast cancer to the lungs.

## Introduction

In the United States, breast cancer will affect approximately one in eight women, with an estimated 229,060 new cases of invasive breast cancer expected to be diagnosed in 2011 and 39,920 deaths [1]. Metaplastic carcinoma of the breast is a rare, quite aggressive form of cancer with a high recurrence rate. This entity accounts for less than 1% of all breast cancers [2]. Due to its high likelihood of recurrence, primary metastatic metaplastic breast cancer is usually treated with combining surgery, chemotherapy, and radiation [2]. Here, we present a case report of a uniquely rapid manifestation of recurrent metaplastic breast cancer.

## Case Report

**Figure 1:**
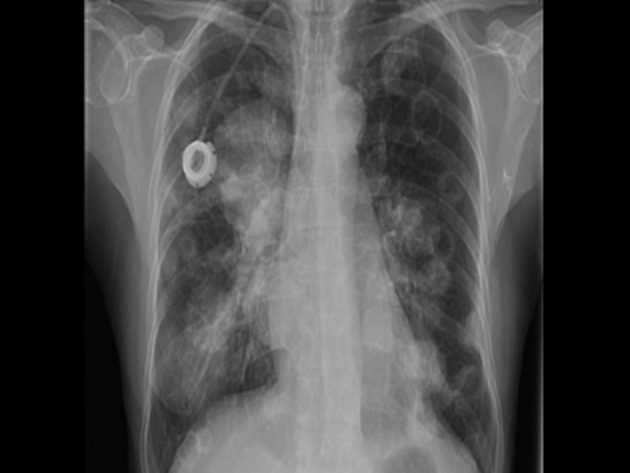
Chest X-ray showing new multiple bilateral cavitary nodules and focal infiltrates in the lateral aspect of the right upper lobe.

A previously healthy 62-year-old Asian American female presented at the City of Hope Cancer Center for evaluation of an enlarging breast lump and an accompanying BIRADS category 2 mammogram. Further radiologic studies, including MRI and ultrasound of the breast revealed a 3.5 cm × 3.5 cm × 2.3 cm mass, with no evidence of systemic metastases. Biopsy of the mass revealed the metaplastic breast cancer (MBC) with spindle cell features and with triple negative (estrogen, progesterone, and HER2/neu receptor negative) phenotype. She was started on neoadjuvant chemotherapy consisting of docetaxel 60 mg/m^2^ given intravenously on day 1, and leucovorin 500 mg/m^2^, cisplatin 25 mg/m^2^, and 5-fluorouracil 500 mg/m^2^ each infused over 24 hours for four days and to be repeated for 3 cycles. However, she progressed after one cycle. She underwent mastectomy with axillary node dissection with the final pathology revealing a T3 lesion and no evidence of lymph node diagnosis. She then received four cycles of adjuvant doxorubicin 60 mg/m^2^ and cyclophosphamide 600 mg/m^2^ and then started local-regional radiation therapy. However, after one week of radiation (1,800 cGy) she developed a fever, and complained of cough, shortness of breath, and fatigue. Chest X-ray revealed new multiple bilateral cavitary nodules and focal infiltrates in the lateral aspect of the right upper lobe (Fig. 1). The differential diagnosis included opportunistic, possible fungal infection, however metastatic disease could no be ruled out. On computerized tomography scan (CT) of the chest consolidation in the lateral right upper lobe measuring 4cm was seen, and, bilateral well-defined rounded lung nodules measuring up to 2.7 cm were described, with several of them demonstrating central cavitation. Multiple new small mediastinal nodes noted, measuring up to 1.1 cm in the pretracheal region. CT-guided biopsy was performed and confirmed the presence of clusters and pools of malignant singe cells present with spindle cell features that were morphologically compatible with the previously known MBC. Bronchial cultures were obtained and were negative for fungi and bacteria. She was started on capecitabine 1,250 mg/m^2^ twice daily orally on 2 weeks on, one week off schedule. After 2 weeks of capecitabine there was evidence of rapid progression and decline of her condition. The patient opted to discontinue treatment and received comfort measures only. She expired two months after being diagnosed with metastasis.

## Discussion

MCB is a histologically diverse neoplasm in which adenocarcinoma coexists with an epithelial or mesenchymal cell population. There are five variants of metaplastic carcinoma: spindle cell, squamous cell, carcinosarcoma, matrix-producing, and metaplastic carcinoma with osteoclastic giant cells [3]. Metaplastic carcinoma of the breast is generally seen in women over the age of fifty, and more likely to occur in African Americans (14%) and is least likely in Asian women (2.7%), and is least likely in Asian women (2.7%). Over 70% of women with MCB present with tumors larger than 2 cm and 20% have tumors larger than 5 cm in size. Despite the large tumor size and poorly or undifferentiated features at presentation in 67.8% of patients at presentation, less than 25% of the patients present with lymph node involvement. The estrogen and progesterone receptors are frequently negative (89% and 90% of the tumors respectively). Of the patients with local, node-negative disease 35% to 62% have recurrence within 2 to 5 years of initial diagnosis compared to 17% to 20% for infiltrating ductal carcinoma (IDC). Metastasis to the lungs is the most common, seen in 31-70% of patients with MBC [1]. Median survival with metastatic disease is 8 months from diagnosis using conventional treatment with chemotherapy and radiation [3].

Surgical treatment for primary MCB mirrors that of IDC, with lumpectomies for appropriate patients and mastectomy for tumors larger than 5 cm [4]. As with IDC, there is no difference in overall survival in patients with MCB treated with mastectomy versus lumpectomy [5, 6]. Most patients with MCB, however, receive mastectomies since they generally present with larger tumors compared to their ICD counterparts [2, 5].

Radiation has been shown to improve overall survival of patients with MCB if administered in the adjuvant setting. Tseng and colleagues reported that adjuvant radiation is beneficial to patients with 4 or more axillary lymph nodes, large primary tumors (≥ 5 cm), and chest wall invasion. However, there was no radiation benefit to patients with tumors ≤ 5 cm and fewer than 4 axillary lymph nodes, indicating that radiation should be a component of multimodality therapy for these patients [5].

Current treatment with chemotherapy for primary and metastatic MCB parallels that of IDC, especially since MCB tends to be triple negative [7], however, survival is poorer than for patients with IDC [8, 9]. We attempted to treat this patient with a platinum-containing modified regimen in the neoadjuvant setting, and followed up by standard adjuvant anthracycline and cyclophosphamide, without success. Since MCB tends to be estrogen, progesterone negative hormonal therapy is generally just as ineffective as chemotherapy.

The patient presented with a fast growing MCB with a spindle cell carcinoma, a rare variant seen in less than 10% of all metaplastic carcinomas and resembling a low-grade sarcoma. The prognosis of spindle cell breast cancers is poor with a 5-year survival rate at around 65%, which is slightly better than survival rates for most metaplastic breast cancers. The prognosis depends on the relative amount spindle cells versus ductal carcinoma elements. Spindle cell breast cancer tumors with a more even split of spindle cell and ductal carcinoma elements are more aggressive and have a poorer prognosis. The most common site of metastasis is the lungs, followed by the bone, and the liver. The average survival time after diagnosis is between 11 and 18 months. There is no standard treatment for spindle cell breast cancer; therefore it parallels that of IDC.

This case is unique for the exceptionally fast metastasis and the unusual radiological findings. The multiple cavitary lung masses associated with fevers are indicative of fungal infections and rarely suggestive of breast carcinoma. Cavitary lung metastases can occur in other tumors, such as sarcomas [10, 11]. The rapid progression to metastases is this case further confirms that traditional chemo- and hormonal therapies for IDC are ineffective against MBC. This case emphasizes the need for personalized, real time assessment of targetable molecular characteristics, since clinical trials investigating novel chemotherapeutic strategies are difficult to conduct due to the rarity of cases like the one we report here.
